# Do hospitalizations push households into poverty in India: evidence from national data

**DOI:** 10.12688/f1000research.145602.1

**Published:** 2024-03-21

**Authors:** Shyamkumar Sriram, Muayad Albadrani

**Affiliations:** 1 Department of Social and Public Health, Ohio University, Athens, Ohio, 45701, USA; 2 Department of Family and Community Medicine, Taibah University, Medina, Al Madinah Province, Saudi Arabia

**Keywords:** Poverty, Out-of-Pocket Health Expenditures, India, Healthcare

## Abstract

**Introduction:**

High percentage of OOP (Out-of-Pocket) costs can lead to poverty and exacerbate existing poverty, with 21.9% of India’s 1.324 billion people living below the poverty line. Factors such as increased patient cost-sharing, high-deductible health plans, and expensive medications contribute to high OOP costs. Understanding the poverty-inducing impact of healthcare payments is essential for formulating effective measures to alleviate it.

**Methods:**

The study used data from the 75th round of the National Sample Survey Organization (Household Social Consumption in India: Health) from July 2017-June 2018, focusing on demographic-socio-economic characteristics, morbidity status, healthcare utilization, and expenditure. The analysis included 66,237 hospitalized individuals in the last 365 days. Logistic regression model was used to examine the impact of OOP expenditures on impoverishment.

**Results:**

Logistic regression analysis shows that there is 0.2868 lower odds of experiencing poverty due to OOP expenditures in households where there is the presence of at least one child aged 5 years and less present in the household compared to households who do not have any children. There is 0.601 higher odds of experiencing poverty due to OOP expenditures in urban areas compared to households in rural areas. With an increasing duration of stay in the hospital, there is a higher odds of experiencing poverty due to OOP health expenditures. There is 1.9013 higher odds of experiencing poverty due to OOP expenditures if at least one member in the household used private healthcare facility compared to households who never used private healthcare facilities.

**Conclusion:**

In order to transfer demand from private to public hospitals and reduce OOPHE, policymakers should restructure the current inefficient public hospitals. More crucially, there needs to be significant investment in rural areas, where more than 70% of the poorest people reside and who are more vulnerable to OOP expenditures because they lack coping skills.

## Introduction

The protection of households from financial risks that are associated with medical expenses is one of the key objectives of health systems. ^1^ India is actively taking steps to offer its population universal health coverage (UHC). As the core of UHC, financial protection is regarded as essential. India is ranked third in the Southeast Asian region in terms of “countries with highest OOP expenditure on health,” according to the World Health Organization. OOP costs are people’s direct payments to healthcare providers at the time-of-service usage, according to the World Health Organization (WHO). ^2^ OOPs are defined as completely private transactions (payments made by patients to private physicians and pharmacies), official patient cost-sharing (user fees/copayments) within predetermined public or private benefit packages, and informal payments (payments made in excess of the prescriptions covered by benefits, both in cash and in kind). OOPs can therefore occur through market transactions, as an explicit part of a policy, or both. OOP health spending may rise whenever families choose to utilize and receive healthcare services, but they are not shielded from high expenses since healthcare is expensive. ^3^

OOP costs make up around 62.6% of all health spending in India, which is one of the highest percentages in the world. ^4^ In India, OOP health costs account for a sizeable amount of total household spending, which inadvertently drives down spending on other essential items and lowers household wellbeing overall. In contrast to the “health for all” idea that was more prevalent in the previous decade, the present policy discussion is about “health for all with financial protection”. ^5^ The National Health Policy 2017 of India places a high priority on affordability and the reduction of financial risk. ^6^ In order to improve fair financing, it is not thought that OOP healthcare payments are a good way to pay for medical services. OOP payments are not a cost-effective way to pay for healthcare, and they may have a negative impact on equity and push vulnerable people into poverty. ^7^ High OOP medical costs can deplete savings and ruin credits, negatively affect medication adherence, quality of life, and other health outcomes. ^8^ OOP payments that are relatively significant have the potential to put vulnerable households into poverty and exacerbate the poverty of households who are already poor.

According to the 2011 World Bank Poverty Line of USD 1.90, around 21.9% of India’s 1.324 billion people live below the poverty line. ^9^ High OOP health costs have an impact on household finances and cause many to fall into poverty, according to the evidence. ^10^ According to a research conducted in India, OOP healthcare payments caused 2.2% of the population to live below the poverty level. ^11^ OOP health costs in India cause the impoverishment of up to 39 million people each year. ^12^^,^^13^ The incidence and depth of poverty can indeed be increased by OOP health expenses, according to the data. Furthermore, poverty has a detrimental effect on one’s health. ^14^ The most significant methods used by households to cover costs include selling assets and borrowing money. OOP healthcare payments worsen both the occurrence and degree of poverty. ^15^ In India, majority of health insurance plans solely cover only hospitalization costs. ^16^

An expected situation in a country with a good health financial protection system is that no one should be forced into poverty as a result of incurring medical costs because of healthcare utilization. ^17^^,^^18^ While some households may spend a higher percentage of their income on healthcare, others may spend a much smaller percentage of their income on healthcare and still be considered to be pushed below the poverty line. According to a recent World Bank Group analysis, OOP payments made up a sizeable portion of all healthcare costs in Central and Eastern European nations. Additionally, patients in poor nations pay out-of-pocket for healthcare treatments at a rate of half a trillion dollars annually (about $80 per person). ^19^ Unfortunately, the poor suffered greatly as a result of these costs.

It becomes more challenging for the Poor People’s Health Insurance Program to offer coverage to households that enter poverty if more people are made poorer by OOP expenses. Even those households who are not particularly near to the poverty threshold may become impoverished as a result of OOP expenses. It becomes challenging for the insurance programs for the poor to remain adaptable enough to permit frequent entry and exit without involving significant administrative expenditures due to the dynamic character of poverty. Additionally, understanding how OOP health spending affects poverty is essential for formulating effective measures to alleviate it. The impact of OOP health expenditures on poverty and the numerous factors that influence the occurrence of poverty are the primary research questions this study would focus on. These are the specific inquiries: (i) What steps may be taken to prevent poverty from spreading? And (ii) what are the variables influencing the incidence of household poverty brought on by OOP medical expenses in India? Using the most recent Social Consumption and Health Survey from the National Sample Survey Organization (NSSO), this study makes an effort to determine how OOP payments affect poverty in India. ^20^ Implementing strong policies can shield households from the frequent and high costs of the healthcare system due to a lack of resources. This analysis is valuable for formulating policies and programs to fight poverty in India, specifically to develop methods for reducing financial risk.

## Methods

### Data source

The study employed national representative unit-level cross-sectional data from the 75th round of the *National Sample Survey Organization (Household Social Consumption in India: Health).* The survey was conducted under the stewardship of the *Ministry of Statistics and Programme Implementation*, Government of India during the time period of July 2017-June 2018. The survey schedule collects the information pertaining to the *demographic*- *socio-economic characteristics*, *morbidity status*, *utilization* of *healthcare services* and *healthcare expenditure* across *ambulatory, inpatient, delivery and immunization* care for households and individuals. A two-stage stratified random sampling design was adopted in the survey with census villages and urban blocks as the First Stage Units for rural and urban areas respectively and households as the Second Stage Units. Overall sample size consisted of the 1,13,823 households and 5,57,887 individuals (including the death cases). The analysis however, circumscribed 66,237 individuals who were hospitalized in the last 365 days of the survey (without childbirth episodes). The detailed information on the survey design can be found in the official report released by the National Sample Survey Organization.

### Factors affecting incidence of impoverishment due to OOP health expenditures

To study the effects of various factors on the occurrence of impoverishment due to OOP health expenditures, the logistic regression model will be used. The logistic regression model is preferred since the dependent variable is dichotomous. “Whether a household falls below poverty line after making OOP healthcare payments?” will be used as the dependent variable. A dichotomous variable for impoverishment will be created with 0 for not falling below poverty line after making OOP healthcare payments and 1 for falling below poverty line after making OOP healthcare payments. Thus, the dichotomous variable created for incidence of impoverishment in the household will serve as the dependent variable for the logistic regression model. The independent variables include the various characteristics of the households.

## Results

Descriptive statistics of the sample are shown in Table 1 which shows that 47.34% of households reported presence of at least one child (aged 5 years and less) in the household. 30.54% of households reported presence of at least one elderly person (aged 60 years and above) in the household. 41.85% of households reported presence of at least one secondary educated member in the households. 55.54% of households were located in the rural areas and 44.46% of households were located in in urban areas. Majority, 20.54% of households reported socioeconomic status of household as lowest income quartile and 19.64% of households reported socioeconomic status of household as highest income quartile. 44.09% of households were small (1-4 members). 47.01% of households were medium (5-8 members) and 8.90% of households were large (9 and more). 37.02% of households reported that at least one member in the household used a private healthcare facility for hospitalization.

**Table 1.** Descriptive statistics of categorical and continuous variables.

Variables	Definitions and categories	Frequency (%)	Weighted percentage (%)
Age groups (children)	Presence of at least one child (aged 5 years and less) in the household	47.34%	33.25%
Age groups (elderly)	Presence of at least one elderly person (aged 60 years and above) in the household	30.54%	29.87%
Marital status	Presence of someone in the household	23.62%	21.23%
Female education	Presence of at least one secondary educated member in the household	41.85%	41.35%
Location of household	Rural	55.54%	66.90%
	Urban	44.46%	33.10%
Socioeconomic status of household	Lowest income quartile	20.54%	30.05%
	Second income quartile	19.27%	22.01%
	Third income quartile	20.87%	21.87%
	Fourth income quartile	19.68%	15.60%
	Highest income quartile	19.64%	10.47%
Drinking water	Safe water	97.66%	99.70%
	Unsafe water	2.34%	1.10%
Household cooking fuel	Unclean fuels	52.90%	6.05%
	Clean fuels	47.10%	39.7%
Drainage type	Open (kutcha and pucca)	41.83%	39.01%
	Covered (pucca and underground)	28.41%	27.91%
	No drainage	29.76%	33.08%
Latrine type	Service and pit latrine	21.84%	17.18%
	Septic tank/flush system	46.80%	41.05%
	No latrine and others	31.30%	41.77%
Household size	Small household (1-4 members)	44.09%	54.91%
	Medium household (5-8 members)	47.01%	41.07%
	Large household (9 and more)	8.90%	4.02%
Religion of the household	Hinduism	77.01%	81.20%
	Islam	14.01%	13.04%
	Christianity	5.86%	2.75%
	Other religions	3.12%	3.01%
Social group of the household	Scheduled tribes	13.01%	9.01%
	Scheduled castes	15.09%	18.98%
	Other backward castes	40.89%	45.54%
	Others	31.01%	26.47%
Level of care of hospitalization	If at least one member in the household used a private healthcare facility for hospitalization	37.02%	10.03%

Variables	Definition	Mean	Standard Error
Sex	Proportion of female members in the household	0.4901	0.0017
Health Insurance coverage	Proportion of members enrolled in health insurance in each household	0.1703	0.0035
Chronic Illness	Proportion of members suffering from chronic illness in each household	0.0653	0.0013
Hospitalization	Proportion members hospitalized in each household	0.0501	0.0006
Duration of hospitalization	Total duration of hospitalization of all members in the household	1.3001	0.02500
Duration of ailment	Total duration of ailment of all members in each household	412.095	11.901
Monthly consumption expenditure	Total consumption expenditure of all members in each household	37,878.30	304.9445
Monthly inpatient OOP health expenditure	Total inpatient OOP health expenditure of all members in each household	245.5302	10.4567
Monthly outpatient OOP health expenditure	Total outpatient OOP health expenditure of all members in each household	121.098	8.238854
Total monthly OOP health expenditure	Total OOP health expenditures of all members in each household	398.089	14.0199

Table 2 shows the incidence of poverty. 15.95% of total households reported poverty which increased to 18.89% after making OOP payments. In rural areas, the incidence of poverty was 18.90% which increased to 21.90 after making OOP payments. In urban areas, the incidence of poverty was 9.30% which increased to 11.30 after making OOP payments. The incidence of poverty in lowest income quartile was 66.98% which increased to 71.03% after making OOP payments. The incidence of poverty was 17.34% for less than 5 days duration of stay in hospital which increased to 18.89% after making OOP payments, while the incidence of poverty was 13.23% for more than 20 days duration of stay in hospital which increased to 32.73% after making OOP payments. The incidence of poverty was 11.23% for at least one member in the household used private healthcare facility which increased to 26.23% after making OOP payments while the incidence of poverty was 18.02% when no member in the household used private healthcare facility which increased to 19.02% after making OOP payments. The incidence of poverty was 24.23% when at least one child aged 5 years and less in the household which increased to 28.01% after making OOP payments. The incidence of poverty was 17.65% where at least one elderly person aged 60 years and above in the household which increased to 20.93% after making OOP payments.

**Table 2.** Incidence of poverty by demographic and household characteristics.

Variables	Categories	Incidence of poverty in population (%)	Incidence of poverty among poor people (%)	Incidence of poverty after making OOP payments in population (%)	Incidence of poverty after making OOP payments among poor people (%)
Percentage of total households reporting poverty		15.95	100	18.89	100
Sector	Rural	18.90	81.62	21.90	81.32
	Urban	9.30	18.38	11.30	18.68
Socioeconomic status of the household	Lowest income quartile	66.98	92.52	71.03	82.01
	Second lowest income quartile	4.98	7.45	12.01	11.78
	Third income quartile	0.00006	0.000083	3.02	0.0324
	Fourth income quartile	0	0	0	0.00143
	Highest fifth income quartile	0	0	0	0.0112
Household size	Small household	9.45	31.02	10.95	31.99
	Medium household	24.01	60.55	25.02	59.00
	Large household	34.00	8.43	28.09	9.01
Religion of the household	Hinduism	15.83	82.01	19.14	81.54
	Islam	19.01	14.23	22.01	15.23
	Christianity	14.95	3.10	17.23	2.05
	Other religions	14.67	0.66	15.36	1.18
Social group of the household	Scheduled tribes	32.13	18.23	34.23	16.23
	Scheduled castes	23.23	26.02	26.12	27.23
	Other backward classes	16.23	39.62	19.01	41.23
	Others	9.03	16.13	12.44	15.31
Duration of stay in hospital	Less than 5 days	17.34	94.89	18.89	88.61
	5-10 days	14.23	2.21	29.89	7.01
	11-20 days	14.34	1.87	32.78	3.26
	More than 20 days	13.23	1.03	32.73	1.12
Private healthcare facility for hospitalization	If atleast one member in the household used private healthcare facility	11.23	7.23	26.23	14.23
	No member in the household used private healthcare facility	18.02	92.77	19.02	85.77
Child aged 5 years and less in the household	At least one child aged 5 years and less in the household	24.23	47.01	28.01	46.91
	No child aged 5 years and less in the household	13.97	52.99	15.21	53.09
Elderly people aged 60 years and above	At least one elderly person aged 60 years and above in the household	17.65	29.21	20.93	31.12
	No elderly people aged 60 years and above in the household	16.23	70.79	19.23	68.88
Secondary educated female in household	At least one secondary educated female in household	9.56	21.12	23.14	31.23
	No secondary educated female in household	21.43	78.88	19.12	68.77
Divorced person in household	At least one divorced person in household	19.02	26.01	21.12	26.23
	No divorced person in household	18.09	73.99	20.23	73.77

Table 3 shows the intensity of poverty due to OOP health expenditures. The poverty gap increased from 19.45% to 23.01% after making OOP payments. In rural areas, the intensity of poverty before making OOP payments was 19.01% which increased to 21.01% after making OOP payments while in the urban areas, the intensity of poverty before making OOP payments was 20.01% which increased to 21.01% after making OOP payments. The intensity of poverty before making OOP payments in lowest income quartile was 21.01% which increased to 23.01% after making OOP payments while in the highest fifth income quartile it increased from 0% to 41.01% after making OOP payments. The intensity of poverty before making OOP payments was 19.34% for less than 5 days duration of stay in hospital which increased to 22.34% after making OOP payments and the intensity of poverty before making OOP payments was 22.13% for more than 20 days duration of stay in hospital which increased to 42.01% after making OOP payments. The intensity of poverty before making OOP payments was 19.43% if at least one member in the household used private healthcare facility which increased to 33.67% after making OOP payments.

**Table 3.** Intensity of poverty by demographic and household characteristics.

Variables	Categories	Pre-OOP payment poverty gap (%)	Post-OOP payment poverty gap (%)
Normalized poverty gap		19.45	23.01
Sector	Rural	19.01	21.01
	Urban	20.01	
Socioeconomic status of the household	Lowest expenditure quartile	21.01	23.01
	Second lowest expenditure quartile	6.23	19.03
	Third expenditure quartile	1.56	31.02
	Fourth expenditure quartile	0	34.14
	Highest fifth expenditure quartile	0	41.01
Household size	Small household	17.01	22.03
	Medium household	20.01	23.01
	Large household	23.01	27.45
Religion of the household	Hinduism	19.75	23.45
	Islam	18.04	27.03
	Christianity	17.06	23.45
	Other religions	22.03	26.01
Social group of the household	Scheduled tribes	24.23	25.23
	Scheduled castes	20.23	24.34
	Other backward classes	18.15	22.02
	Others	17.25	22.13
Duration of stay in hospital	Less than 5 days	19.34	22.34
	5-10 days	18.98	31.23
	11-20 days	21.23	37.23
	More than 20 days	22.13	42.01
Private healthcare facility for hospitalization	If atleast one member in the household used private healthcare facility	19.43	33.67
	No member in the household used private healthcare facility	19.23	22.41
Child aged 5 years and less in the household	At least one child aged 5 years and less in the household	19.83	23.45
	No child aged 5 years and less in the household	19.23	22.67
Elderly people aged 60 years and above	At least one elderly person aged 60 years and above in the household	18.78	23.43
	No elderly people aged 60 years and above in the household	19.78	22.52
Secondary educated female in household	At least one secondary educated female in household	17.56	22.63
	No secondary educated female in household	19.67	23.23
Divorced person in household	At least one divorced person in household	18.01	23.76
	No divorced person in household	18.98	22.98

Table 4 shows the logistic regression analysis results for the incidence of poverty due to OOP health expenditures. Logistic regression analysis shows that there is 0.2868 lower odds of experiencing poverty due to OOP expenditures in households where there is the presence of at least one child aged 5 years and less present in the household compared to households who do not have any children. There is 0.601 higher odds of experiencing poverty due to OOP expenditures in households in urban areas compared to households in rural areas. There is a decrease in the odds of experiencing poverty due to OOP expenditures in households with an increase in household income. Both medium and larger households have lower odds of incurring poverty due to OOP health expenditures compared to smaller households. With an increasing duration of stay in the hospital, there is a higher odds of experiencing poverty due to OOP health expenditures with an odds of 1.4013 of experiencing poverty due to OOP expenditures for a 5-10 duration of stay in hospital compared to other stay durations compared to 13.9702 higher odds of experiencing poverty due to OOP expenditures for more than 20 days duration of stay in hospital. There is 1.9013 higher odds of experiencing poverty due to OOP expenditures if at least one member in the household used private healthcare facility compared to households who never used private healthcare facilities for treatment. There is 4.6781 higher odds of experiencing poverty due to OOP expenditures if the household has members who suffer from chronic illnesses.

**Table 4.** Logistic regression results for the incidence of impoverishment due to OOP health expenditures.

Incidence of poverty after making OOP payments	Odds ratio	95% Confidence Interval	P
Presence of at least one child aged 5 years and less present in the household	0.7132	0.6740 – 0.9027	0.001
Presence of at least one elderly aged 60 years and above present in the household	1.0831	0.8601 – 1.3189	0.890
Presence of someone divorced in the household	1.1234	0.7638 – 1.3456	0.197
Sector			
Rural (reference)			
Urban	1.6010	1.4234 – 1.7234	0.000
Socioeconomic status			
Poorest income quartile (reference)			
Second lowest income quartile	0.8207	0.1324 – 0.2345	0.0000
Third income quartile	0.0512	0.0301 – 0.0645	0.0000
Fourth income quartile	0.0231	0.0112 – 0.3932	0.0000
Highest fifth income quartile	0.0093	0.0065 – 0.0234	0.0000
Household size			
Small household (reference)			
Medium household (5-8)	0.6907	0.5656 – 0.8378	0.000
Large household (9 or more)	0.4967	0.3891 – 0.6331	0.000
Duration of stay in hospital			
Less than 5 days			
5-10 days	2.4013	2.1016 – 2.9785	0.000
11-20 days	4.9014	4.1234 – 6.0980	0.000
More than 20 days	14.9702	10.987 – 19.876	0.000
At least one member in the household used private healthcare facility	2.9013	1.9896 – 3.4352	0.000
Proportion of female members in the household	1.0623	0.6457 – 1.1101	0.987
Proportion of members with chronic illness in each household	5.6781	0.3452 – 9.8901	0.000
At least one member is covered by insurance	0.7235	0.5734 – 0.8345	0.000
Constant	1.6545	0.8890 – 3.0987	0.301

Also, most importantly, there is a 0.2765 lower odds of experiencing poverty due to OOP expenditures if at least one member is covered by health insurance, highlighting the importance of health insurance coverage in protecting households from impoverishment due to OOP health expenditures.

## Discussion

Our studies indicate that the population’s overall poverty rate was 18.89% after OOP payments, and that the normalized poverty difference among households that were already poor due to OOP medical expenses widened by 3.06%. Our findings fall short of World Bank estimates, according to which 21.9% of the population lived below the poverty line, calculated using the updated World Bank Poverty Line. ^21^ Another research that used NSS data for the years 1995–1996 indicated that 2.2% of the population was living in poverty as a result of OOP health expenses. ^22^ The results we have obtained indicate that after making OOP payments, the prevalence of poverty has increased among households belonging to various SES levels. According to the results of the logistic model, all households in all other quintiles of expenditure have lower probability of being poor than the poorest families. The likelihood of being financially poor as a result of OOP health expenses decreased steadily as the socioeconomic status of the households rose, with the richest households having the lowest probabilities. This result is in line with other research that has been published in the literature. ^23^^,^^24^

Our study showed that the presence of at least one child aged 5 years and less present in the household increases the incidence of poverty after making OOP payments. Studies carried out in Ethiopia also demonstrated that presence of at least one child aged 5 years and less present in the household increases the incidence of poverty after making OOP payments. ^25^ Our study showed that households in urban areas experienced increased incidence of poverty after making OOP payments compared to rural areas. Research carried out in low-income countries like Uganda, Malawi and Nigeria showed that households in urban areas experienced increased the incidence of poverty after making OOP payments compared to rural areas. ^26^ Similar studies in Ethiopia found that households in urban areas experienced increased the incidence of poverty after making OOP payments compared to rural areas. ^27^ Our studies show that households in lowest income quartiles experienced increased incidence of poverty after making OOP payments compared to higher income quartiles. Research on this issue demonstrated that households in lowest income quartiles experienced increased incidence of poverty after making OOP payments compared to higher income quartiles. ^28^ Another study in Kenya found that households in lowest income quartiles experienced increased incidence of poverty after making OOP payments compared to higher income quartiles. ^29^ Similar study carried out in Uganda showed that low-income households experienced increased incidence of poverty after making OOP payments compared to their higher income counterparts. ^30^

Our study showed that medium and large sized households experienced increased the incidence of poverty after making OOP payments compared to small sized households. Findings from a study done in Turkey found that household size played an important role in increased incidence of poverty after making OOP payments. ^31^ A study in India found that medium and large sized households experienced increased the incidence of poverty after making OOP payments compared to small sized households. ^32^ Similar study in India found that medium and large sized households experienced increased the incidence of poverty after making OOP payments. ^33^ Our study shows that with increased duration of stay in hospital increases the incidence of poverty after making OOP payments. Other research carried out in India showed that with increased duration of stay in hospital increases the incidence of poverty after making OOP payments. ^34^ Another study in India found that with increased duration of stay in hospital increases the incidence of poverty after making OOP payments. ^35^ Similar study in Bangladesh found that duration of hospital stay has profound impact on increasing the incidence of poverty after making OOP payments. ^36^ Our study showed that the presence of at least one member in the household who used private healthcare facilities increases the incidence of poverty after making OOP payments. A study carried out in Kenya showed that using private healthcare facility increases the incidence of poverty after making OOP payments. ^29^ Another study carried out in Tajikistan found that using private healthcare facility increases the incidence of poverty after making OOP payments. ^37^ Similar study in Turkey found that using private healthcare facility increases the incidence of poverty after making OOP payments. ^38^

Our studies show that the proportion of members with chronic illness in each household increases the incidence of poverty after making OOP payments. A study carried out in India demonstrated that number of members with chronic illness in each household increases the incidence of poverty after making OOP payments. ^39^ Another study in Nepal found that a greater number of members with chronic illness in each household increases the incidence of poverty after making OOP payments. ^40^ Further research on this issue in low middle income countries found that family members with chronic illness increases the incidence of poverty after making OOP payments. ^41^ Our study showed that presence of at least one member is covered by insurance increases the incidence of poverty after making OOP payments compared to members not covered by insurance.

It comes to light that long-term illness plays a significant role in predicting poverty brought on by OOP payments. According to studies, hospitalizations are also significantly influenced by chronic conditions. ^42^ In our analysis, the odds of poverty were more than twice as high for households with at least one member suffering from a chronic illness than they were for homes without such a member. Further studies conducted on this issue came to the same conclusion that chronic diseases are key factors driving households into poverty; the location of hospitalization, whether in a public or private institution, has an impact on health expenses. ^43^^,^^44^ Around 49% of all available beds are in the private sector, which is home to a sizable network of unregulated private hospitals in India. ^45^ Our research demonstrated that a person’s hospitalization location also affects whether or not OOP payments will cause them to become impoverished.

A study in Turkey found that presence of at least one member is covered by insurance increases the incidence of poverty after making OOP payments compared to members not covered by insurance. ^38^ Another study in Ghana found that members with insurance experienced increased the incidence of poverty after making OOP payments compared to members not covered by insurance. ^46^ Similar study in Nigeria found that hat members with insurance experienced increased the incidence of poverty after making OOP payments compared to members not covered by insurance. ^47^

Our research demonstrates that, both in urban and rural areas, the prevalence of poverty has increased as a result of OOP payments. The proportion of persons who become poor after completing OOP payments has grown in urban regions relative to rural ones, although only slightly. This is because only poor people are taken into account. This demonstrates how OOP health expenses are higher for urban residents, which forces them into poverty. The results of the logistic regression also indicate that households in urban regions are more likely than those in rural areas to become impoverished as a result of OOP health expenses. According to evidence from the literature, the number of urban poor people is constantly growing as a result of poor people moving from rural areas to cities in pursuit of economic possibilities. The majority of these migrants settle in crowded city slums with subpar living circumstances. ^48^ According to the most recent census, 33% of Indians reside in urban settings, and 250 million more will do so by the year 2030. In this group, 27% of urban residents are below the poverty line. ^49^

Our estimations may have been skewed by the increased migration from rural to urban regions in quest of employment. Although there is a benefit to living in an urban area in terms of access to health care, most urban poor people do not have access to this benefit. ^50^ The National Rural Health Mission was established by the GOI in 2005 to address the health needs of the rural population. The National Urban Health Mission was not established by the government until 2014 to assist the urban poor, strengthen the health infrastructure in urban areas, and lower OOP health expenditures. ^51^ The absence of political will to address the health needs of the urban poor is demonstrated by the delay in developing the urban health program for the underprivileged. The National Urban Health Mission and other programs meant to mitigate the OOP burden of the urban population are not performing successfully, as seen by the higher likelihood of poverty among the urban population. Additionally, the majority of urban poor people who depend on daily income are unable to be admitted to hospitals, which may hinder their ability to go to work. However, none of the current health insurance plans available give coverage for outpatient care; the present health insurance programs for the poor only cover hospitalization. Due to the present health insurance programs for the poor’s lack of coverage for outpatient care and their desire to avoid losing their jobs, they may be forced to pay OOP, which will raise their costs. Additionally, compared to households with fewer individuals, larger households are less likely to become impoverished as a result of OOP health expenses. Larger families may be able to arrange for a member of the family to serve as a caretaker in the event of illness or incapacity, which is one of the most likely causes. Furthermore, many common ailments may not require hospitalization because of this family caregiving. Evidence from the United States demonstrates that home health care services have cut down on hospital visits and length of stays. ^52^

In India, 400 million people live on less than $1.25 per person per day, and according to our data, these poor people spend 11–15% of their total income on OOPHE on average. ^53^ OOPHE accounts for a substantial percentage of total spending, hence governments should offer health insurance to the poor to lessen their susceptibility to both health and economic shocks. The use of private clinics for medical treatment is the second factor contributing to the high OOPHE. ^54^ In India, private facilities account for more than 75 percent of health expenditures. Because of their superior health infrastructure and higher level of service, private institutions are preferred over public ones **.** In order to transfer demand from private to public hospitals and reduce OOPHE, policymakers should restructure the current inefficient public hospitals. More crucially, there needs to be significant investment in rural areas, where more than 70% of the poorest people reside and who are more vulnerable to CHE because they lack coping skills.

## Data Availability

The data from the National Sample Survey Organization (NSSO) of the Government of India was used for the study. Social Consumption (Health), NSS 75 ^th^ Round for 2017-2018 was used for this analysis. The survey covered whole of the Indian Union. Data can be obtained from the Government of India or from the corresponding author by reasonable request.
