# Supplementary material for: Do hospitalizations push households into poverty in India: evidence from national data
Source: F1000Res. 2024 Mar 21;13:205. [Version 1] doi: 10.12688/f1000research.145602.1 (PMC11007365; doi:10.12688/f1000research.145602.1)
Supplement: Peer review history

# Reviewer report: Reviewer response for version 1

**DOI:** 10.5256/f1000research.159573.r261034

**Published:** 2024-05-15

**Authors:** Olaide Sekinat Opeloyeru

**Affiliations:** 1 Tai Solarin University of Education, Ijebu Ode, Ogun State, Nigeria

1. The introduction is attractive to read but the authors need to pass the message of the write up through existing literature. There is no trace of past research related to this work in India. I am aware that a lot of work has been done in India which people have referenced. The authors need to insert a section separately on the literature review as well to show the gap filled in the existing literature. As it is, it is difficult to see the new contribution to existing literature.
2. The sub-heading under the methodology “Factors affecting incidence of impoverishment due to OOP health expenditures” should be removed and the authors should avoid the use of future words like “will” since the work has been done and is not a proposal (an example of this is under methodology second paragraph, line 2.) The authors should explain how each variable was partitioned or designed for their study.
3. Appropriate statistics were used but the interpretation and how data were reported should be fine-tuned. For example some statistics were reported in 3 decimal places and others in 4 decimal places, there should be consistency. Also, the interpretation of logistic regression should be improved on for readers to appreciate your work (for example, health services received through OOP in the urban area increases the chance or odds of living below the poverty by 60% compared to rural area which is in contrast to what you put in your paper as “There is 0.601 higher odds of experiencing poverty due to OOP expenditures in households in urban areas compared to households in rural areas”).
4. The authors joined discussion of results with the conclusion. A separate heading should be created for conclusions and appropriate language should be used. For example, where the author used “coping skills”, I want to suggest the author use “coping strategies”

On a general note, appropriate language and good editorial work is needed for this study e.g. “Similar study in Nigeria found that hat members with insurance experienced increased the incidence of poverty after making OOP payments compared to members not covered by insurance”.

Is the work clearly and accurately presented and does it cite the current literature?

Partly

If applicable, is the statistical analysis and its interpretation appropriate?

Partly

Are all the source data underlying the results available to ensure full reproducibility?

Yes

Is the study design appropriate and is the work technically sound?

Partly

Are the conclusions drawn adequately supported by the results?

Partly

Are sufficient details of methods and analysis provided to allow replication by others?

Yes

Reviewer Expertise:

NA

I confirm that I have read this submission and believe that I have an appropriate level of expertise to confirm that it is of an acceptable scientific standard, however I have significant reservations, as outlined above.

# Reviewer report: Reviewer response for version 1

**DOI:** 10.5256/f1000research.159573.r261053

**Published:** 2024-04-10

**Authors:** Sumedh Bele

**Affiliations:** 1 University of Oxford, Oxford, England, UK

Thank you for the opportunity to review this research article – Do hospitalizations push households into poverty in India: evidence from national data.

 This study makes valuable addition to the literature around the impact of out of pocket (OOP) medical expenditures on poverty. Authors have appropriately analysed large dataset to demonstrate the impact of OOP on poverty. I just have few minor comments to further strengthen this article:

 - I would recommend authors to add citations for few key statements in the introduction section such as, :India is actively taking steps to offer its population universal health coverage (UHC)", "India is ranked third in the Southeast Asian region in terms of “countries with highest OOP expenditure on health,” according to the World Health Organization".

 - In its current format it is a little hard to understand key take home message from this study. So, I would suggest authors to carve out a "conclusion" section which provides succinct conclusion of this study.

Is the work clearly and accurately presented and does it cite the current literature?

Yes

If applicable, is the statistical analysis and its interpretation appropriate?

I cannot comment. A qualified statistician is required.

Are all the source data underlying the results available to ensure full reproducibility?

Yes

Is the study design appropriate and is the work technically sound?

Yes

Are the conclusions drawn adequately supported by the results?

Yes

Are sufficient details of methods and analysis provided to allow replication by others?

Yes

Reviewer Expertise:

public health and health services research

I confirm that I have read this submission and believe that I have an appropriate level of expertise to confirm that it is of an acceptable scientific standard.

# Reviewer report: Reviewer response for version 1

**DOI:** 10.5256/f1000research.159573.r261054

**Published:** 2024-04-05

**Authors:** Seetharaman Narayanan

**Affiliations:** 1 Professor and Head of Community Medicine, KMCH Institute of Health Sciences and Research, Coimbatore, Tamilnadu, India

**Assessment:**

 1. Presentation and Literature Review:

 The article is well-structured and clearly presented. It provides a comprehensive review of relevant literature, highlighting the importance of the research problem and situating the study within the existing body of knowledge.

 2. Study Design and Academic Merit:

 The study design is appropriate for addressing the research questions. The use of nationally representative survey data from the NSSO enhances the generalizability and academic merit of the findings.

 3. Methods and Repeatability:

 The methods section provides sufficient details on the data source, variables, and analytical techniques (logistic regression) to allow for replication by others. A short discussion on the suitability of the World bank definition of poverty line (over any other definition of poverty line) would be helpful.

 4. Statistical Analysis:

 The statistical analysis appears appropriate, with logistic regression models employed to examine the factors influencing the incidence of poverty due to OOPHE. The interpretation of the results is generally sound.

 5. Data Availability:

 The authors mention that the data can be obtained from the Government of India or by reasonable request. It would be preferable to provide more specific information on data accessibility to enhance transparency and reproducibility.

 6. Conclusions and Support:

 The conclusions drawn are generally supported by the results presented. The authors highlight the crucial role of health insurance coverage in protecting households from impoverishment due to OOPHE and the need for policy interventions to improve access to public healthcare facilities, particularly in rural areas.

**Strengths:**

 - Nationally representative data enhances generalizability.

 - Comprehensive literature review and sound methodology.

 - Highlights the role of health insurance and public healthcare access in alleviating poverty due to OOPHE.

**Suggestions:**

 - Clarify the applicability of the World Bank defined poverty line (vis-a-vis any other methodology to define the poverty line currently in use, in India) in the analysis.

 - Provide more specific information on accessing the source data data from NNSO, to enhance reproducibility.

 - Discuss potential limitations and directions for future research.

 - Expand on the policy implications and recommendations for addressing the identified issues.

 Overall, the article makes a valuable contribution to understanding the impoverishing effects of OOPHE in India and identifying factors that influence household poverty.

Is the work clearly and accurately presented and does it cite the current literature?

Yes

If applicable, is the statistical analysis and its interpretation appropriate?

Yes

Are all the source data underlying the results available to ensure full reproducibility?

Yes

Is the study design appropriate and is the work technically sound?

Yes

Are the conclusions drawn adequately supported by the results?

Yes

Are sufficient details of methods and analysis provided to allow replication by others?

Yes

Reviewer Expertise:

Public Health, Health Economics

I confirm that I have read this submission and believe that I have an appropriate level of expertise to confirm that it is of an acceptable scientific standard.
